# Clinical Significance of a Multicancer Screening Trial With Stage-Based End Points

**DOI:** 10.1001/jamanetworkopen.2025.36247

**Published:** 2025-10-09

**Authors:** Kemal Caglar Gogebakan, Jane Lange, Lukas Owens, Amalie Pinderup, Roman Gulati, Larry G. Kessler, Georgios Lyratzopoulos, Ruth Etzioni

**Affiliations:** 1 Division of Public Health Sciences, Fred Hutchinson Cancer Center, Seattle, Washington; 2 Cancer Early Detection Advanced Research Center, Knight Cancer Institute, Oregon Health & Science University, Portland; 3 Epidemiology of Cancer Healthcare & Outcomes Group, Department of Behavioural Science, Institute of Epidemiology and Health Care, University College London, London, United Kingdom; 4 Department of Health Systems and Population Health, School of Public Health, University of Washington, Seattle

## Abstract

**Importance:**

The first randomized screening trial of a multicancer early detection test is ongoing, with the primary end point being the incidence of late-stage cancer. The unprecedented use of a stage-based end point and short 3-year follow-up raise questions about how results should be interpreted and used in developing multicancer screening policy.

**Objective:**

To estimate outcomes of the trial and to identify information from the estimates that may aid in interpreting results of short-term trials evaluating multicancer screening tests.

**Design, Setting, and Participants:**

This multicancer decision-analytic model estimated outcomes from registry data from the England National Cancer Registration and Analysis Service for cases diagnosed between January 2013 and December 2018. This model simulated a population-based multicancer screening trial of average-risk participants without a prior cancer diagnosis. The analysis was performed between April 2024 and April 2025.

**Interventions:**

Three annual multicancer screenings at months 0, 12, and 24. Cancers were assumed detectable 1 or 2 years before clinical diagnosis based on a published analysis, and cancer-specific early-stage sensitivities were set to either 100% or 50% of published sensitivities among clinically diagnosed cases.

**Main Outcomes and Measures:**

Reductions in late-stage (stage III-IV) cancer incidence over 3 years, cancer mortality over 5 years, and contributions of each cancer type to reductions in late-stage incidence and cancer mortality across the range of detectable intervals and early-stage sensitivities.

**Results:**

The model simulated 70 000 participants per arm (screening and control; median age, 66 years), and estimated that the overall late-stage incidence reductions at 3 years ranged from 6% to 23%, with corresponding reductions in 5-year cancer mortality from 6% to 9%. Colorectal cancer contributed the most to the reduction in late-stage incidence (28% to 39%), while lung cancer contributed the most to mortality reduction (40% to 42%).

**Conclusions and Relevance:**

This independent decision-analytic model study found that the trial could achieve nontrivial cancer downstaging over 3 years, but modest mortality reduction over 5 years. These results were due to a limited number of target cancer types, underscoring the importance of transparent reporting of outcomes by cancer type, consideration of mortality implications, and careful preplanning for subsequent evaluation steps by the cancer research community.

## Key Points

**Question:**

How should the scientific community judge the clinical significance of the first randomized multicancer screening trial with its stage-based end point?

**Findings:**

This independent decision-analytic model study with 2 simulated cohorts of 70 000 participants estimated that the trial may achieve a 6% to 23% reduction in late-stage incidence over 3 years and a 6% to 9% reduction in cancer mortality over 5 years, with lung and colorectal cancers contributing the most to these reductions.

**Meaning:**

These estimates challenge the research community to weigh the clinical significance of short-term, stage-based end points and to recognize the importance of sufficiently detailed reporting of test performance and outcomes by cancer type.

## Teaser

This decision analytic model estimates outcomes of a randomized multicancer screening trial with stage-based end points and identifies information from the estimates that may aid in interpreting results of short-term trials evaluating multicancer screening tests.

## Introduction

Advances in blood-based cancer biomarkers are poised to usher in a new era of cancer screening, with multicancer early detection (MCED) tests used to screen for multiple cancer types.^1,2,3^ The English National Health Service (NHS)–Galleri screening trial will screen participants with the Galleri MCED test annually for 3 years.^4^ The primary report of the trial results is expected in 2026.^5^

As the first randomized study of an MCED test, the trial is unprecedented. Not only does it evaluate a novel multicancer screening technology, but it is also the first screening trial with late-stage incidence as the primary end point. This end point will be assessed after a short follow-up period of 3 years after randomization.^5^ The established end point for cancer screening trials is cancer mortality (except for screening tests that can lead to preventing disease, where cancer incidence may also be a primary end point).^6^ The time required to observe an association of screening with cancer mortality has necessitated longer follow-up intervals.

In the case of the NHS- Galleri trial, the 3-year reduction in the incidence of late-stage cancer will be the only result concerning screening benefit available at the time of the primary report.^7^ We have little to no experience judging the clinical significance of late-stage incidence results. Screening trials are frequently designed to be able to detect a mortality reduction of 20%.^8,9^ A mortality analysis of the NHS-Galleri trial is planned after 5 years of follow-up from randomization^5^; while this is still short term in relation to other screening trials, it could help to inform the clinical significance of the stage-based finding.

At this point, the expected magnitude of any late-stage reduction in the trial is unclear. An early interim analysis to potentially greenlight a purchase of 1 million tests by the NHS did not result in the purchase taking place.^7,10^ Both meta-analyses of screening trials in different cancers^11,12^ and modeling studies in cancers without trials^13,14^ have indicated that associations of screening with late-stage diagnoses tend to exceed corresponding expected associations with mortality. An alternative stage-based end point^15,16^ aggregates late-stage incidence at and after diagnosis but requires longer follow-up.

Given these uncertainties, it seems prudent to develop a plan now for how to process, interpret, and disseminate the results of short-term multicancer screening trials with incidence-based end points, including the NHS-Galleri trial in particular. What will be considered a clinically significant late-stage incidence reduction at 3 years? Will the associated mortality reduction factor into the interpretation of any late-stage incidence finding? Will it be important to highlight the contributions of specific cancer types? What other information will be needed to judge the validity, generalizability, and actionability of the results? Recent studies have examined whether it might be reasonable to simply use late-stage incidence reduction as a surrogate or alternative to mortality^11,12,17,18^ but have only analyzed cancer types for which screening trials exist.^11,12,13^

We present an independent decision analytic model—distinct from the company-affiliated model that was used in designing the trial^19^—to estimate the trial outcomes as a foundation for a discussion about how such outcomes should be interpreted. We calibrated the model to the trial population and used published information about the performance of the Galleri test at and prior to clinical diagnosis to estimate the 3-year, stage-based outcome. We augmented this with calculations of the associated mortality reduction at 5 years and the cancer type-specific downstaging and mortality reductions. By fast forwarding to the publication of the trial results, we aim to accelerate decisions on how to transparently report and interpret outcomes from short-term MCED trials to best inform the evidence concerning multicancer screening benefits.

## Methods

### Modeling Framework

#### Natural History Model

We adapted a previously reported model of cancer initiation, progression, and detection, developed to address the lack of evidence regarding the likely benefit of MCED tests that target cancer types without existing screening programs.^20^ The model partitions disease natural history into early- and late-stage MCED, test-detectable, preclinical, and clinical disease states. Estimation of the state transition rates requires inputting the duration of the detectable interval prior to clinical diagnosis and the duration of the preclinical late-stage interval. Lange et al^20^ referred to these inputs as overall mean sojourn time (OMST) and late-stage mean sojourn time (LMST), respectively, and we use the same acronyms here; they showed that specification of the OMST and LMST permits estimation of the early-stage mean sojourn time, which underlies reduction in late-stage incidence under screening given test sensitivities for early- and late-stage disease sensitivity for each target cancer.

#### Mortality Model

Owens et al^13^ presented a framework to estimate the mortality benefit based on the reduction in late-stage cancer incidence. In their model, cases that would have been diagnosed as late-stage cancer without screening but were diagnosed in early stage by screening (ie, stage-shifted cases) received an extension of their time to disease-specific death based on survival differences between clinically diagnosed early- and late-stage cases.

#### Multicancer Framework

We adapted the multicancer model for the average-risk population in England and used it to estimate late-stage incidence and cancer mortality in the screen and control arm for each cancer type. This approach allows individuals to have multiple cancer types and assumes they continue multicancer screening after their first diagnosis. It also assumes risks of preclinical onset and diagnosis for each cancer type are independent of these risks for other cancer types. For details of the framework, see the eMethods in Supplement 1.

### Data Sources and Model Inputs

We modeled the outcomes of the trial for 11 of the 12 cancer types included in the trial design (anus, bladder, colon or rectum, esophagus, head and neck, liver or bile duct, lung, lymphoma, ovary, pancreas, and stomach). Myeloma and plasma cell neoplasm was also used in the trial design, but we excluded it because it is not stageable. We also did not model breast, prostate, and other cancer types because they were not included among the 12 cancer types used in the trial design. We used publicly available, cancer-specific incidence data from the England National Cancer Registration and Analysis Service (NCRAS) for cases diagnosed between January 2013 and December 2018. Stage distributions (TNM stages I-IV and unknown) and 5-year net survival estimates for TNM stages I to IV, categorized by cancer type, were derived from a population-based MCED modeling study^17^ using NCRAS data for individuals aged 50 to 79 years diagnosed between January 2013 and December 2018. We defined early stage as TNM stages I and II and late stage as TNM stages III and IV. For each cancer, we calculated the stage distribution by early- and late-stage disease and estimated 5-year net survival for early- and late-stage disease using incidence-weighted averages. We did not explicitly model background screening utilization, but the observed incidence rates from the NCRAS reflect existing screening utilization in England. For details on stage-specific incidence and survival data sources, see the eMethods in Supplement 1. Stage distributions by cancer type in the absence of MCED screening are provided in eTable 1 in Supplement 1, and 5-year net survival estimates by cancer type and stage in eTable 2 in Supplement 1.

To specify the OMST, we used information from the only study to date of the NHS-Galleri test sensitivity prior to clinical diagnosis.^21^ This stored-serum study showed rapidly declining test sensitivity with time prior to clinical diagnosis (eg, 15.2% and 6% of cases were detectable 1 year and 2 years before diagnosis, respectively, and this fraction increased for cases who later died of their disease). Given these results, we assumed very little detection more than 2 years prior to clinical diagnosis and set the OMST for the modeled test to either 1 or 2 years. The study did not provide cancer type–specific sensitivities, but these sensitivities for clinically diagnosed cases are available from a different large-scale study (eTable 3 in Supplement 1).^22^

We assumed that the LMST ranged from 6 months to 1 year across all cancer types based on studies of increases in medical care utilization prior to cancer diagnosis in the UK.^23,24^ We further assumed that late-stage sensitivities were similar to those observed in clinically diagnosed, late-stage cases during this time. However, we considered that early-stage sensitivity in the prospective MCED screening trial may be lower than that observed among clinically diagnosed, early-stage cases, as suggested by prior prospective studies that reported lower sensitivity in screening compared with clinical diagnosis settings.^1,25^ Based on these considerations, we modeled 3 scenarios, which we labeled based on the triplet of OMST, LMST, and early-stage test sensitivity: (1) fast-fast-optimistic (OMST of 1 year and LMST of 6 months with early-stage sensitivity per cancer set to 100% of the published values), (2) slow-fast-conservative (OMST of 2 years and LMST of 6 months with early-stage sensitivity set to 50% of the published values), and (3) slow-slow-conservative (OMST of 2 years and LMST of 1 year, also with early-stage sensitivity set to 50% of the published values).

### Modeled Screening Trial and Estimated Outcomes

The median age of participants in the trial is 66 years.^26^ Following the trial design (summarized in the eMethods in Supplement 1), we simulated a trial enrolling 70 000 individuals per arm. We assumed 100% adherence to screening across the 3 rounds and that all positive tests are followed by perfectly accurate confirmatory assessment. For each scenario, we estimated the cancer-specific and combined cumulative reduction in late-stage incidence up to 1 year after the last screen. We also estimated the cancer-specific and combined cumulative mortality reduction up to 3 years after the last screen. This study did not require institutional review board review because it was considered non–human participants research in accordance with Common Rule. Analysis followed the noncost aspects of the Consolidated Health Economic Evaluation Reporting Standards (CHEERS) reporting guideline.^27^

### Statistical Analysis

The model was built and analyzed in R version 4.1.3 software (R Project for Statistical Computing). Because this was a decision-analytic modeling study, no formal significance testing was conducted; model uncertainty was assessed varying the natural history parameters and the test sensitivities. Analysis was conducted from April 2024 to April 2025.

## Results

The model was based on 140 000 simulated participants (70 000 per arm; median age, 66 years). The model estimated the trial outcomes under 3 scenarios.

### Combined Late-Stage Incidence

**Figure 1.**
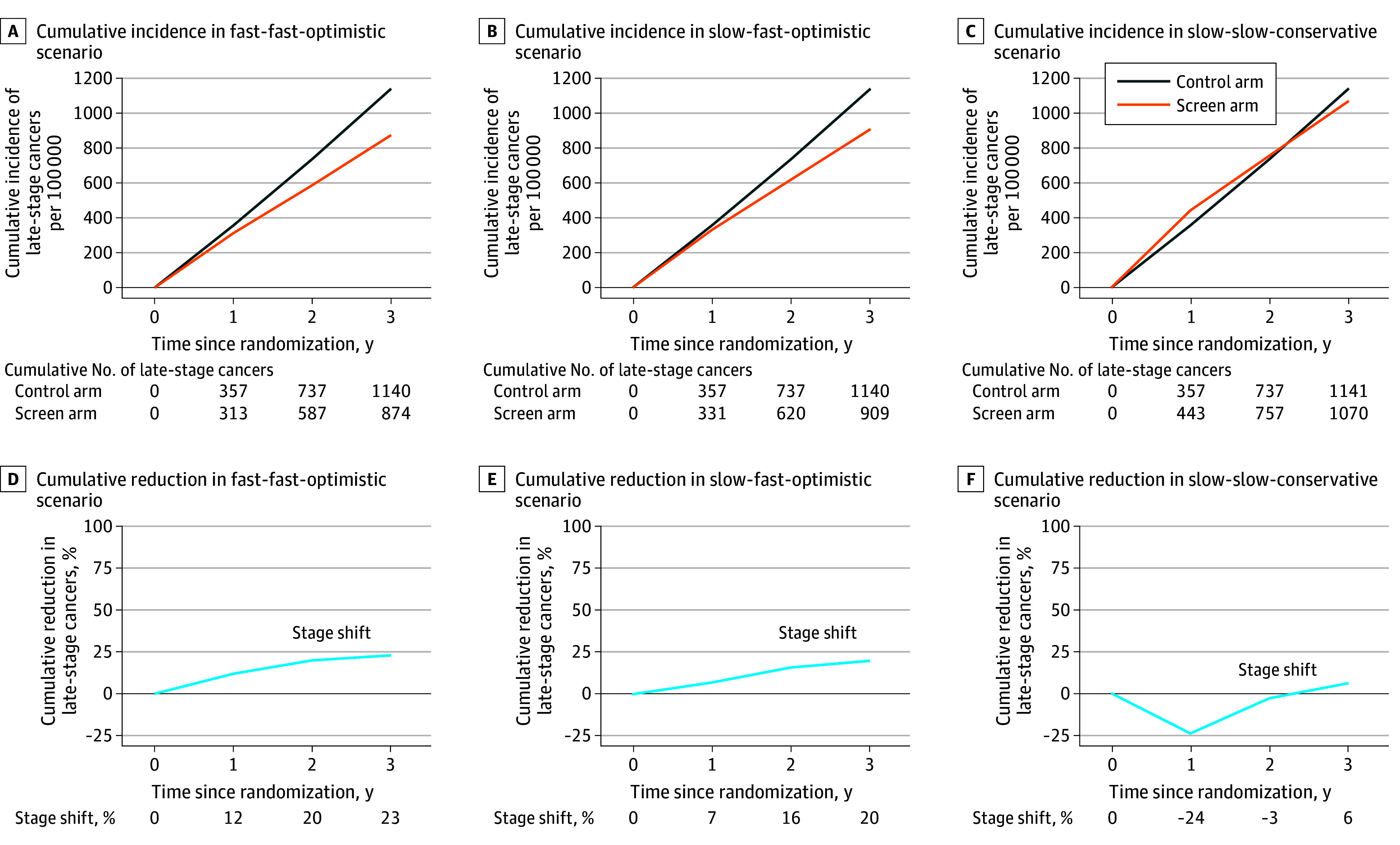
Estimated Cumulative Late-Stage Incidence per Arm and Cumulative Reduction in Late-Stage Incidence in a Simulated National Health Service–Galleri Trial Estimates assume a screening trial with 3 annual screens (months 0, 12, and 24) starting at age 66 years and 1 year of follow-up after the last (third) screen. Results are presented under 3 scenarios, which were labeled based on the triplet of overall mean sojourn time (OMST), late-stage mean sojourn time (LMST), and early-stage test sensitivity. (1) The fast-fast-optimistic scenario (panels A and D) had an OMST of 1 year and an LMST of 6 months with early-stage sensitivity per cancer set to 100% of the published estimates by Klein et al.^22^ B, E. (2) The slow-fast-conservative scenario (panels B and E) had an OMST of 2 years and an LMST of 6 months with early-stage sensitivity set to 50% of the published estimates. (3) The slow-slow-conservative scenario (panels C and F) had an OMST of 2 years and an LMST of 1 year with early-stage sensitivity set to 50% of the published estimates.

Figure 1 presents the cumulative late-stage incidence estimates for both arms, along with estimates of combined reduction in late-stage incidence under the specified scenarios. The estimated cumulative late-stage incidence in the control arm was 1140 cases per 100 000. In the screening arm, under scenario 1 and 2, the model estimated 23% and 20% cumulative reduction in late-stage incidence, respectively. Under scenario 3, the model estimated an initial increase in late stage until 1 year of follow-up after the second screening round, and a late-stage incidence reduction of 6% at the end of 3 years.

### Combined Cancer Mortality

**Figure 2.**
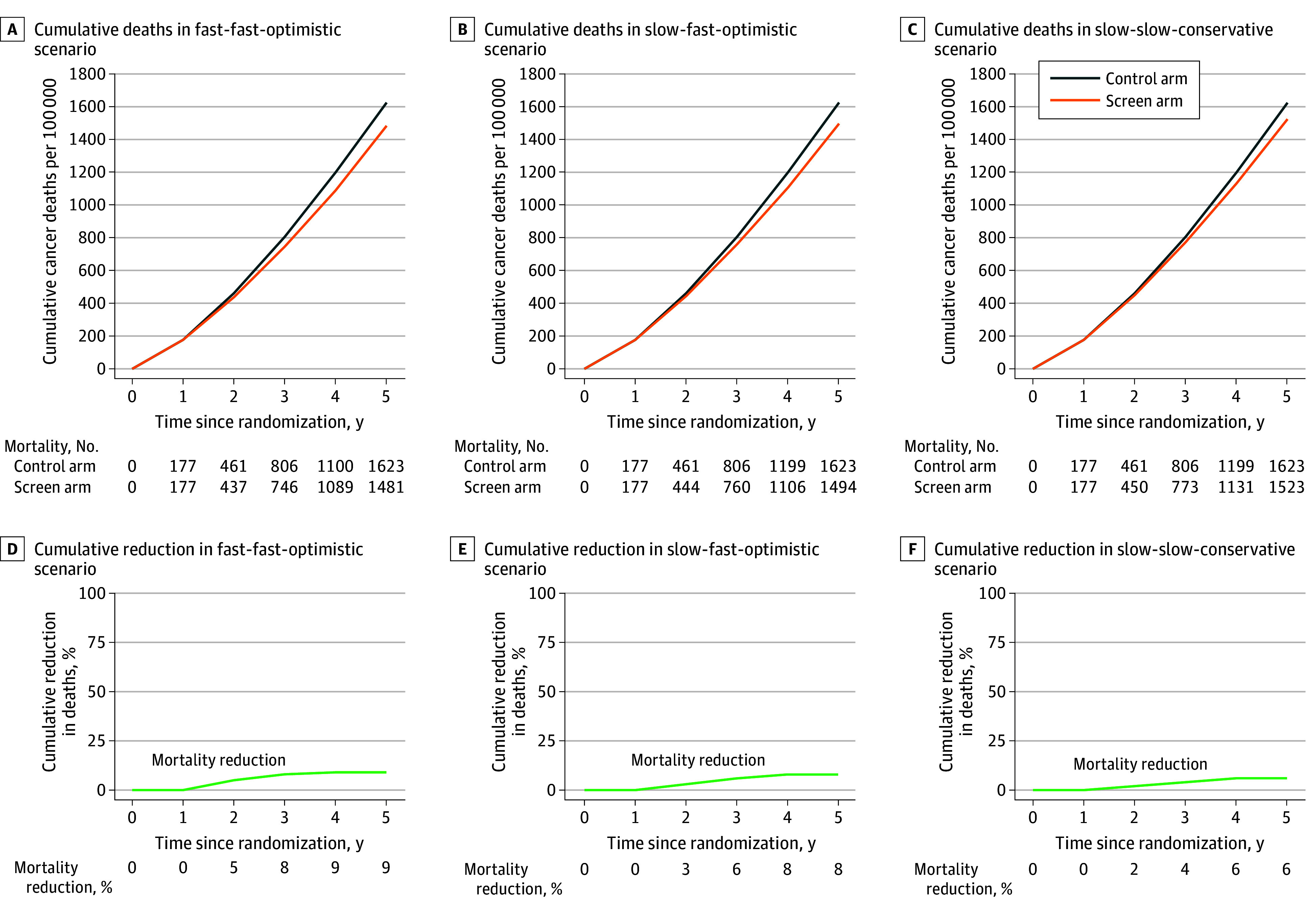
Estimated Cumulative Cancer Deaths per Arm and Relative Reduction in Cumulative Cancer Deaths in a Simulated National Health Service–Galleri Trial Estimates assume a screening trial with 3 annual screens (years 0, 1, and 2) starting at age 66 years and 3 years of follow-up after the last (third) screen. Results are presented under 3 scenarios, which were labeled based on the triplet of overall mean sojourn time (OMST), late-stage mean sojourn time (LMST), and early-stage test sensitivity. (1) The fast-fast-optimistic scenario (panels A and D) had an OMST of 1 year and an LMST of 6 months with early-stage sensitivity per cancer set to 100% of the published estimates by Klein and colleagues.^22^ (2) The slow-fast-conservative scenario (panels B and E) had an OMST of 2 years and an LMST of 6 months with early-stage sensitivity set to 50% of the published estimates. (3) The slow-slow-conservative scenario (panels C and F) had an OMST of 2 years and an LMST of 1 year with early-stage sensitivity set to 50% of the published estimates.

Figure 2 shows the estimated cumulative cancer deaths for both arms, along with the estimated combined cancer mortality reduction under the specified scenarios. For the 11 cancer types, the estimated number of cancer deaths in the control arm was 1623 per 100 000. In the screening arm, under scenarios 1, 2, and 3, the model estimated 9%, 8%, and 6% combined cancer mortality reduction over 5 years, respectively.

### Relative Reductions in Late-Stage Incidence and Cancer Mortality by Cancer Type

Table 1 shows the estimated relative reductions in late-stage incidence by cancer type, with the largest reductions observed in liver and bile duct cancers (scenario 1: 41%; scenario 2: 34%; scenario 3: 18%) and colorectal cancer (scenario 1: 31%; scenario 2: 25%; scenario 3: 11%). Table 1 also shows the estimated mortality reductions, which were highest for colorectal (scenario 1: 16%; scenario 2: 11%; scenario 3: 14%), ovarian (scenario 1: 16%; scenario 2: 11%; scenario 3: 14%), and liver and bile duct cancers (scenario 1: 14%; scenario 2: 6%; scenario 3: 12%).

**Table 1.** Estimated Relative Reductions in Cumulative Late-Stage Incidence and Cancer Deaths by Cancer Type in a Simulated National Health Service–Galleri Trial

Cancer type	Relative reduction in cumulative late-stage incidence by scenario, %^a^^,^^b^			Relative reduction in cumulative cancer deaths by scenario, %^a^^,^^c^		
	(1) Fast-fast-optimistic	(2) Slow-fast-conservative	(3) Slow-slow-conservative	(1) Fast-fast-optimistic	(2) Slow-fast-conservative	(3) Slow-slow-conservative
Anus	24	19	6	10	6	8
Bladder	9	6	−4	3	2	2
Colon or rectum	31	25	11	16	11	14
Esophagus	20	17	4	7	8	7
Head and neck	32	27	12	13	10	11
Liver or bile duct	41	34	18	14	6	12
Lung	17	14	2	8	4	7
Lymphoma	21	18	6	6	5	5
Ovary	27	23	9	16	11	14
Pancreas	26	23	9	7	5	7
Stomach	14	12	−1	6	4	5

^a^ Results are presented under 3 scenarios, which were labeled based on the triplet of overall mean sojourn time (OMST), late-stage mean sojourn time (LMST), and early-stage test sensitivity. (1) The fast-fast-optimistic scenario had an OMST of 1 year and an LMST of 6 months with early-stage sensitivity per cancer set to 100% of the published estimates by Klein et al.^22^ (2) The slow-fast-conservative scenario had an OMST of 2 years and an LMST of 6 months with early-stage sensitivity set to 50% of the published estimates. (3) The slow-slow-conservative scenario had an OMST of 2 years and an LMST of 1 year with early-stage sensitivity set to 50% of the published estimates.

^b^ Estimates assume a screening trial with 3 annual screens (months 0, 12, and 24) starting at age 66 years and 1 year of follow-up after the last (third) screen.

^c^ Estimates assume a screening trial with 3 annual screens (months 0, 12, and 24) starting at age 66 years and 3 years of follow-up after the last (third) screen.

### Contribution of Each Cancer Type to the Combined Reductions in Late-Stage Incidence and Cancer Mortality

Table 2 and eFigure 1 in Supplement 1 show that colorectal cancer contributed the most to the estimated reduction in late-stage incidence, ranging from 28% to 39%, followed by lung cancer at 8% to 24% and head and neck cancer at 11% to 15%. Together, 3 three cancer types accounted for 62% to 64% of the combined cumulative reduction in late-stage incidence. The cancer types contributing the least—anus, bladder, and stomach—accounted for 1% to 4% of the reduction in late-stage incidence.

**Table 2.** Relative Contributions of Each Cancer to the Estimated Reductions in Combined Cumulative Late-Stage Incidence and Cancer Deaths in a Simulated National Health Service–Galleri Trial

Cancer type	Relative contribution to reduction in combined cumulative late-stage incidence by scenario, %^a^,^b^			Relative contribution to reduction in combined cumulative cancer deaths by scenario, %^a^^,^^c^		
	(1) Fast-fast-optimistic	(2) Slow-fast-conservative	(3) Slow-slow-conservative	(1) Fast-fast-optimistic	(2) Slow-fast-conservative	(3) Slow-slow-conservative
Anus	1	1	1	0	0	0
Bladder	1	1	0	1	1	1
Colon or rectum	29	28	39	21	21	20
Esophagus	6	6	4	6	7	6
Head and neck	11	11	15	6	5	5
Liver or bile duct	6	6	10	9	8	9
Lung	24	25	8	40	40	42
Lymphoma	8	7	9	2	2	2
Ovary	4	4	4	3	3	3
Pancreas	8	9	10	9	10	9
Stomach	2	2	0	3	3	3

^a^ Results are presented under 3 scenarios, which were labeled based on the triplet of overall mean sojourn time (OMST), late-stage mean sojourn time (LMST) and early-stage test sensitivity. (1) The fast-fast-optimistic scenario had an OMST of 1 year and LMST of 6 months with early-stage sensitivity per cancer set to 100% of the published estimates by Klein et al.^22^ (2) The slow-fast-conservative scenario had an OMST of 2 years and an LMST of 6 months with early-stage sensitivity set to 50% of the published estimates. (3) The slow-slow-conservative scenario had an OMST of 2 years and an LMST of 1 year with early-stage sensitivity set to 50% of the published estimates. ^b^Estimates assume a screening trial with 3 annual screens (months 0, 12, and 24) starting at 66 years of age and 1 year of follow-up after the last (third) screen. In descending order of contribution: colon or rectum, lung, head and neck, pancreas, lymphoma, liver or bile duct, esophagus, ovary, stomach, anus, bladder.

^c^ Estimates assume a screening trial with 3 annual screens (months 0, 12, and 24) starting at 66 years of age and 3 years of follow-up after the last (third) screen. In descending order of contribution: lung, colon or rectum, pancreas, liver or bile duct, esophagus, head and neck, stomach, ovary, lymphoma, bladder, anus.

Table 2 and eFigure 2 in Supplement 1 show that lung cancer contributed the most to the estimated cumulative mortality reduction at 5 years after randomization, ranging from 40% to 42%, followed by colorectal cancer at 20% to 21%, and pancreatic cancer at 9% to 10%. Together, these three cancer types accounted for 70% to 71% of the combined cancer mortality reduction. The least contributing cancers—anus, bladder, and lymphoma—accounted for 3% of the cancer mortality reduction.

## Discussion

As the first randomized trial of a MCED technology, the NHS-Galleri trial has the potential to influence recommendations concerning this and other MCED tests. This decision analytic model estimated a plausible range for the trial results to facilitate discussion of how to judge the clinical significance of stage-based end points. The model estimated that the NHS-Galleri trial could reduce late-stage incidence by 6% to 23% over 3 years and cancer mortality by 6% to 9% for the targeted cancers over 5 years.

While reduction in late-stage incidence may be of value in and of itself, there is no guideline for determining clinical significance of a specific late-stage incidence reduction in a multicancer setting with absent information about mortality. Our estimated range of 5-year cancer mortality reductions is narrower and more modest than the range of 3-year late-stage reductions. We caution, however, that mortality reductions in screening trials generally deepen over time and may be underestimated by short-term results. It seems likely that more information will be needed to determine the clinical significance of any short-term reduction in late-stage incidence.

In a trial where staging is key, the exact protocol for staging should be clearly spelled out and uniformly applied in both arms, and data on timing and type of staging diagnostics used on screening and control arms should be made available for transparency. This information, plus information on patterns of diagnosis (interval vs screen), stage at diagnosis, and treatment in both screen and control arms will be needed for modeling outcomes over extended time intervals and in different populations. This information could help to estimate disease natural history across cancer types. These estimates will, in turn, facilitate studying different testing protocols and even MCED products.

A standard criterion for success of a decision-analytic model is that it provides accurate estimates. To produce a model that is most likely to be accurate, we have carefully selected model parameters that reflect the best current knowledge about detectability and natural history. Even if the model is ultimately shown not to accurately estimate late-stage incidence reduction, it will have been successful if it helps the field to set criteria for clinical significance in MCED trials with late-stage reduction as an end point.

This work is an attempt to stay ahead of the curve by considering at the present time key questions that will need to be addressed when the trial results are published. We focus specifically on the critical issue of clinical significance, which will be an important factor underlying regulatory decisions. Although the criteria for approval vary across regulatory bodies and health systems, in general, a late-stage incidence reduction is a necessary but not sufficient condition for population screening recommendations.

A key implication of the model results is that, while MCED tests are appealing partly because they offer the possibility of detecting cancer types for which screening programs do not currently exist, the rarest of these cancers may not contribute noticeably to any late-stage incidence or mortality reductions. We therefore recommend that late-stage results be reported overall and per targeted cancer type. Our results indicate that a few high-prevalence cancer types could generate most of the benefit, suggesting that even if the tests can detect a wide range of cancer types, they may not all be impacted in practice. Breaking down the aggregate benefits of MCED screening and reporting the contributions by cancer might guide the development of more efficient MCED strategies that prioritize cancers contributing most to overall benefit; this may reduce the number of unnecessary procedures, avoid more complex confirmation pathways, and reduce associated costs.

### Limitations

Our analysis is limited by the natural history model and the data available for key natural history specifications. The natural history model assumes all cancers inevitably progress to clinical diagnosis; for cancer types without existing screening tests, the suitability of this model structure has not been evaluated. Our specification of 1 to 2 years for the OMST may reflect an optimistic interpretation of the single retrospective study^21^ that represents the only available information on performance of the Galleri test prior to clinical diagnosis. The appropriate value for this input could change with advances in MCED technologies. The aforementioned retrospective study^21^ did not differentiate between cancer types. Therefore, we set the OMST to be the same across cancer types. We further assumed similar LMST across cancer types; we expect results will likely fall within the range of the model estimates under the more extreme scenarios considered. The mortality model does not account for differing tumor subtypes among early- and late-stage cases within cancers. Owens et al^14^ examined subtype-specific stage-shift models for ovarian and prostate cancer; their findings suggest that accounting for disease subtypes would not dramatically change conclusions. Even if the model accurately estimates late-stage incidence reduction, it may still misestimate mortality for certain cancer types, such as pancreatic cancer, where stage shift may not reflect survival due to the natural history of the disease and pronounced lead-time and length biases. Furthermore, the mortality model does not accommodate within-stage survival benefits or therapeutic advances for late-stage disease. If utilization rates and benefits of novel treatments by stage are known, this can be incorporated into our mortality model as in our previous work modeling the mortality benefit of lung cancer screening under novel therapies.^28^ If the mortality estimates do not perfectly match the trial results, they will still illustrate the point that properly interpreting the late-state result may be challenging and may require considering mortality implications. For a list of the limitations of the model, see eTable 4 in Supplement 1.

## Conclusions

This independent decision-analytic model found that, based on plausible assumptions about natural history and test performance, the NHS-Galleri trial could achieve a reportable reduction in late-stage cancer incidence, but a modest reduction in cancer-specific mortality. Most of the estimated benefit from multicancer screening was attributable to a few high-prevalence cancers. These findings highlight the importance of carefully interpreting the clinical significance of stage-based end points and of detailed reporting of outcomes from the first randomized multicancer screening trial, which may set a precedent for evaluating MCED tests.

## Notes

Supplement 1. eMethods eTable 1. Observed and adjusted TNM stage distribution (without screening with multi-cancer test) by cancer type, based on crude incidence rates of 50–79-year-old cases diagnosed between 2013 and 2018 in England eTable 2. Five-year net survival (%) by cancer type and TNM stage category, based on 50–79-year-old cases diagnosed between 2013 and 2018 in England. eTable 3. Multi-cancer test sensitivity by cancer type and TNM stage, based on sensitivities reported in the final Circulating Cell-free Genome Atlas substudy evaluating the Galleri® multi-cancer test eFigure 1. Relative contribution of each cancer type (in descending order of contribution) to the estimated combined cumulative reduction in late-stage incidence in a simulated NHS-Galleri® trial eFigure 2. Relative contribution of each cancer type (in descending order of contribution) to the estimated combined cumulative reduction in cancer deaths in a simulated NHS-Galleri® trial eTable 4. Limitations of the decision-analytic model eReferences

Supplement 2. Data Sharing Statement
