# Supplementary material for: Clinical Significance of a Multicancer Screening Trial With Stage-Based End Points
Source: JAMA Netw Open. 2025 Oct 9;8(10):e2536247. doi: 10.1001/jamanetworkopen.2025.36247 (PMC12511994; doi:10.1001/jamanetworkopen.2025.36247)
Supplement: Supplement 1. — eMethods eTable 1. Observed and adjusted TNM stage distribution (without screening with multi-cancer test) by cancer type, based on crude incidence rates of 50–79-year-old cases diagnosed between 2013 and 2018 in England eTable 2. Five-year net survival (%) by cancer type and TNM stage category, based on 50–79-year-old cases diagnosed between 2013 and 2018 in England. eTable 3. Multi-cancer test sensitivity by cancer type and TNM stage, based on sensitivities reported in the final Circulating Cell-free Genome Atlas substudy evaluating the Galleri® multi-cancer test eFigure 1. Relative contribution of each cancer type (in descending order of contribution) to the estimated combined cumulative reduction in late-stage incidence in a simulated NHS-Galleri® trial eFigure 2. Relative contribution of each cancer type (in descending order of contribution) to the estimated combined cumulative reduction in cancer deaths in a simulated NHS-Galleri® trial eTable 4. Limitations of the decision-analytic model eReferences [file jamanetwopen-e2536247-s001.pdf]

# Supplemental Online Content

Gogebakan KC, Lange J, Owens L, et al. Clinical significance of a multicancer screening trial with stage-based end points. *JAMA Netw. Open*. 2025;8(10):e2536247. doi:10.1001/jamanetworkopen.2025.36247

**eMethods**
**eTable 1.** Observed and adjusted TNM stage distribution (without screening with multi-cancer test) by cancer type, based on crude incidence rates of 50–79-year-old cases diagnosed between 2013 and 2018 in England
**eTable 2.** Five-year net survival (%) by cancer type and TNM stage category, based on 50–79-year-old cases diagnosed between 2013 and 2018 in England.
**eTable 3.** Multi-cancer test sensitivity by cancer type and TNM stage, based on sensitivities reported in the final Circulating Cell-free Genome Atlas substudy evaluating the Galleri® multi-cancer test
**eFigure 1.** Relative contribution of each cancer type (in descending order of contribution) to the estimated combined cumulative reduction in late-stage incidence in a simulated NHS-Galleri® trial
**eFigure 2.** Relative contribution of each cancer type (in descending order of contribution) to the estimated combined cumulative reduction in cancer deaths in a simulated NHS-Galleri® trial
**eTable 4.** Limitations of the decision-analytic model
**eReferences**

This supplemental material has been provided by the authors to give readers additional information about their work.

© 2025 Gogebakan KC et al. *JAMA Network Open*.

## eMethods

### 1.1. Multi-cancer Framework

Given the targeted cancer types in our analysis, we use the multi-cancer model for an average-risk population. We estimate late-stage incidence in the screen and control arm for each cancer type and calculate the combined relative reduction in late-stage incidence in the trial at time $T$ as follows:

$$\frac{\sum_j v_c^j(T) - v_s^j(T)}{\sum_j v_c^j(T)} \quad (1)$$

where $v_c^j(T)$ is the late-stage incidence for cancer type $j$ in the control arm, and $v_s^j(T)$ is the late-stage incidence for cancer type $j$ in the screen arm. This approach allows individuals to have multiple cancer types and assumes they continue screening after the first diagnosis. It also assumes risks of preclinical onset and diagnosis for each cancer type are independent of these risks for other cancer types.

For the combined relative cancer mortality reduction in the multi-cancer screening trial, we follow a similar approach:

$$\frac{\sum_j \theta_c^j(T) - \theta_s^j(T)}{\sum_j \theta_c^j(T)} \quad (2)$$

where $\theta_c^j(T)$ is the mortality due to cancer type $j$ in the control arm, and $\theta_s^j(T)$ is the mortality due to cancer type $j$ in the screen arm.

### 1.2. Data Sources

#### 1.2.1. Age and stage-specific incidence

Given the overall mean sojourn time (OMST) and late-stage mean sojourn time (LMST), the five-state natural history model parameters of Lange et al[1] can be uniquely estimated using age- and stage-specific incidence rates from cancer registry data of the relevant population to NHS-Galleri® trial (England). To the best of our knowledge, the National Cancer Registration and Analysis Service (NCRAS) did not publicly report the joint distribution of age- and stage-specific incidence for patients diagnosed during 2013-2018. Instead, they report age-specific incidence separately from stage-specific incidence; the latter is aggregated across patients of all ages. Using the stage distribution for all ages combined is not appropriate for our analysis, as late stage cancer is more common in older patients (particularly those older than 80) and patients were invited for screening (by the NHS-Galleri® trial) at a much younger age. To address this, we followed a series of steps. First, we obtained five-year band age-specific incidence rates up to age 80 from NCRAS for cases diagnosed between 2013 and 2018.[2] Incidence rates per 100,000 persons were provided for all cancers, except for sex-specific cancers (e.g., ovarian cancer), for which sex-specific rates were created by multiplying the overall rate by the proportion of the relevant sex in the corresponding five-year age band. For stage distribution, we used data from Sasieni et al[3], who reported crude incidence rates for the cancer types in our analyses by Stage I-IV (including unknown stage) for individuals aged 50–79 diagnosed between 2013 and 2018 based on population-based national cancer registry data (NCRAS). We categorized Stage I-II as early-stage and Stage III-IV as late-stage. To handle missing stage data, we again followed the approach by Sasieni et al[3], assuming that missingness was completely random and imputing cancers with missing and unknown stage information by using the relative proportions of non-missing stages (see eTable 1). After imputation, we applied the resulting stage distribution uniformly to the five-year band age-specific incidence data obtained from NCRAS to generate approximate five-year age- and stage-specific incidence rates for each cancer type. While this approximation may introduce some limitations for younger age groups, it is unlikely to significantly impact our results, as the NHS-Galleri® Trial enrolls individuals aged 50–77 and does not include younger participants. This dataset was subsequently used to estimate the parameters in Lange and colleagues'[1] model. For further technical details of the model, see Lange and colleagues.[1]

#### 1.2.2. Age- and stage-specific survival

To the best of our knowledge, the NCRAS did not provide joint age- and stage-specific survival estimates prior to 2022. To address this, we used five-year net survival estimates for Stage I–IV cancers diagnosed between 2013 and 2018 in individuals aged 50–79, as reported by Sasieni et al[3] for the cancer types included in our analysis. These

© 2025 Gogebakan KC et al. *JAMA Network Open*.

estimates, based on the NCRAS data, were calculated using a period approach with the Pohar Perme estimator (capped at 99.9%) and were censored on January 5, 2020, ensuring at least one year of follow-up for all patients.

While Sasieni et al[5] reported net survival separately for Stage I–IV, we computed weighted averages for early-stage (defined as Stage I–II) and late-stage (Stage III–IV) net survival using the relative proportions of cases within each group. Specifically, early-stage net survival was calculated as a weighted average of Stage I and II net survival estimates, and late-stage net survival as a weighted average of Stage III and IV estimates, using the imputed stage distribution among individuals aged 50–79 as weights (see eTable 2). Since our screening program begins at age 66—within this age range—we consider this approach appropriate. Ideally, survival estimates for a narrower age band (e.g., 65–69) would be preferable, but this remains a limitation of the study. We then fit an exponential distribution to the net survival estimates for each cancer type for use in our model.

#### 1.2.3. Multi-cancer test sensitivity by cancer site and stage at diagnosis

Klein et al[4] conducted the third and final substudy of the Circulating Cell-free Genome Atlas (CCGA) study (NCT02889978), a prospective, case-controlled observational study designed to evaluate the Galleri® multi-cancer test (MCT). The objective of this substudy was to validate a further refined version of the Galleri® MCT for use as a screening tool. The pre-specified substudy included 4,077 participants in an independent validation set (cancer: n = 2,823; non-cancer: n = 1,254, with non-cancer status confirmed at one-year follow-up). Klein et al[4] reported the sensitivity of the Galleri® MCT by cancer type and TNM Stage I–IV.

To calculate early-stage (Stage I–II) and late-stage (Stage III–IV) sensitivities for each cancer type, we used the stage distribution among cancer-confirmed participants reported in Klein et al[4]. While Klein et al[4] reported sensitivity separately for Stage I–IV, we computed weighted averages for early and late stages using the relative proportions of cases within each group. Specifically, early-stage sensitivity was calculated as a weighted average of Stage I and II sensitivities, and late-stage sensitivity as a weighted average of Stage III and IV sensitivities, with the stage distribution used as weights (eTable 3).

### 1.3 NHS-Galleri® Trial Design

The NHS-Galleri® trial enrolled patients during Aug 2021–Jul 2022.[5] In total, 140,000 participants aged 50-77 years without previous cancer diagnosis and not undergoing investigation for suspected cancers from NHS Cancer Alliance regions of England were ransomised 1:1 to the intervention or control arm. In the intervention arm, blood samples are analyzed using the Galleri® test; results are unblinded only for those with a cancer signal detected, who are then referred to NHS urgent suspected cancer or non-specific symptoms pathways for prompt confirmatory diagnostic assessment. Intervention arm participants without a cancer signal detected, and all control arm participants (whose blood samples are stored for secondary and exploratory analyses), remain blinded throughout the trial. Rather than relying on electronic case report forms, the trial's outcomes data are primarily sourced from linkage with central NHS data sources, primarily the National Cancer Registration and Analysis Service (NCRAS), which is part of NHS England. The three annual screenings will be conducted at months 0, 12, and 24. The primary endpoint, the reduction in stage III and IV (late-stage) cancer incidence in the intervention arm compared to the control arm, will be assessed at one year of follow-up after the last (3rd) screen (three years after randomization).[5] The secondary endpoint, cancer mortality reduction, will be assessed at three years of follow-up after the last (3rd) screen (five years after randomization).[6]

© 2025 Gogebakan KC et al. *JAMA Network Open*.

**eTable 1. Observed and adjusted TNM stage distribution (without screening with multi-cancer test) by cancer type, based on crude incidence rates of 50–79-year-old cases diagnosed between 2013 and 2018 in England.**

| | TNM Stage Distribution (%) | | | | |
|---|---|---|---|---|---|
| | Observed[a] | | | Adjusted[a] | |
| Cancer Type | Early Stage (I-II) | Late Stage (III-IV) | Unknown/ Missing Stage | Early Stage (I-II) | Late Stage (III-IV) |
| Anus | 37 | 45 | 18 | 45 | 55 |
| Bladder | 66 | 23 | 11 | 74 | 26 |
| Colon/Rectum | 41 | 51 | 8 | 45 | 55 |
| Esophagus | 22 | 64 | 14 | 26 | 74 |
| Head and Neck | 29 | 58 | 13 | 33 | 67 |
| Liver/Bile Duct | 14 | 30 | 56 | 32 | 68 |
| Lung | 25 | 68 | 7 | 27 | 73 |
| Lymphoma | 27 | 55 | 18 | 32 | 68 |
| Ovary | 31 | 54 | 16 | 36 | 64 |
| Pancreas | 17 | 61 | 21 | 22 | 78 |
| Stomach | 23 | 58 | 19 | 28 | 72 |

[a] 'Observed' data reflect the observed stage distribution prior to imputing cases with unknown or missing stage. 'Adjusted' data reflect the stage distribution after imputation and were used in outcome estimations for the simulated NHS-Galleri® trial.

© 2025 Gogebakan KC et al. *JAMA Network Open*.

**eTable 2. Five-year net survival (%) by cancer type and TNM stage category, based on 50–79-year-old cases diagnosed between 2013 and 2018 in England. All sexes combined except for ovarian cancer.**

| Cancer Type | Early Stage (I-II) | Late Stage (III-IV) |
|---|---|---|
| Anus | 86 | 52 |
| Bladder | 72 | 24 |
| Colon/Rectum | 90 | 44 |
| Esophagus | 46 | 11 |
| Head and Neck | 84 | 52 |
| Liver/Bile Duct | 41 | 5 |
| Lung | 52 | 6 |
| Lymphoma | 84 | 67 |
| Ovary | 90 | 25 |
| Pancreas | 23 | 2 |
| Stomach | 52 | 11 |

© 2025 Gogebakan KC et al. *JAMA Network Open*.

**eTable 3. Multi-cancer test sensitivity by cancer type and TNM stage, based on sensitivities reported in the final Circulating Cell-free Genome Atlas substudy evaluating the Galleri® multi-cancer test.**

| Cancer Type | Early Stage (I-II) | Late Stage (III-IV) |
|---|---|---|
| Anus | 0·50 | 1 |
| Bladder | 0·18 | 0·83 |
| Colon/Rectum | 0·67 | 0·92 |
| Esophagus | 0·48 | 0·97 |
| Head and Neck | 0·72 | 0·93 |
| Liver/Bile Duct | 0·94 | 1 |
| Lung | 0·40 | 0·93 |
| Lymphoma | 0·46 | 0·66 |
| Ovary | 0·60 | 0·90 |
| Pancreas | 0·62 | 0·88 |
| Stomach | 0·33 | 0·94 |

© 2025 Gogebakan KC et al. *JAMA Network Open*.

**eFigure 1. Relative contribution of each cancer type (in descending order of contribution) to the estimated combined cumulative reduction in late-stage incidence in a simulated NHS-Galleri® trial.** [a]

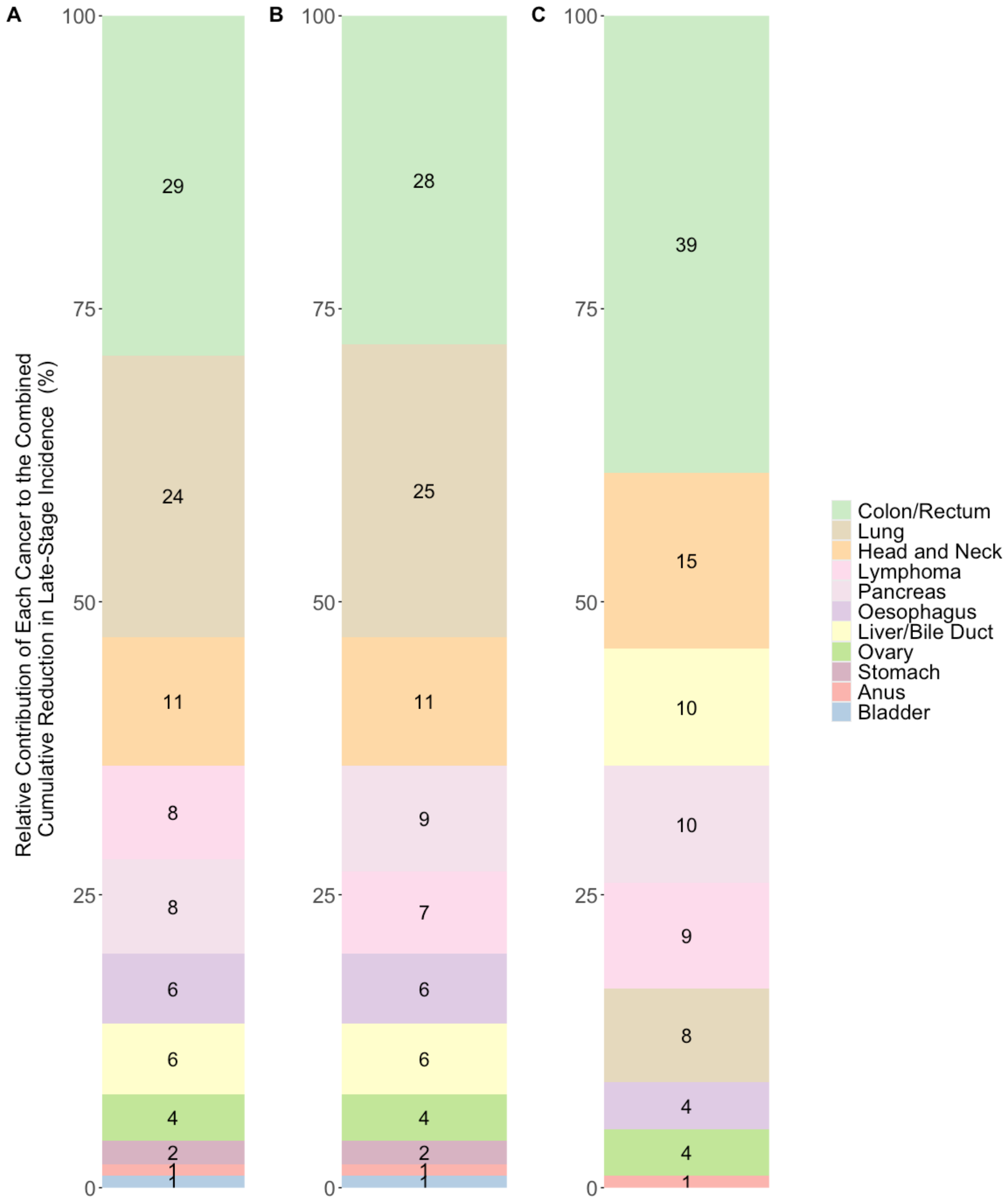


[a]Estimates assume a screening trial with 3 annual screens (months 0, 12, 24) starting at 66 years of age and 1 year of follow-up after the last (3rd) screen. Results are presented at three years after randomization under three scenarios, which we labeled based on the triplet of OMST, LMST and early-stage test sensitivity: A.(1) Fast-fast-optimistic: Overall mean sojourn time (OMST) of 1 year and late-stage mean sojourn time (LMST) of 6 months with early-stage sensitivity per cancer set to 100% of the published estimates by Klein et al[4]; B. (2) Slow-fast-conservative: OMST of 2 years and LMST of 6 months with early-stage sensitivity set to 50% of the published estimates and C. (3) Slow-slow-conservative: OMST of 2 years and LMST of 1 year with early-stage sensitivity set to 50% of the published estimates.

© 2025 Gogebakan KC et al. *JAMA Network Open*.

**eFigure 2. Relative contribution of each cancer type (in descending order of contribution) to the estimated combined cumulative reduction in cancer deaths in a simulated NHS-Galleri® trial.[a]**

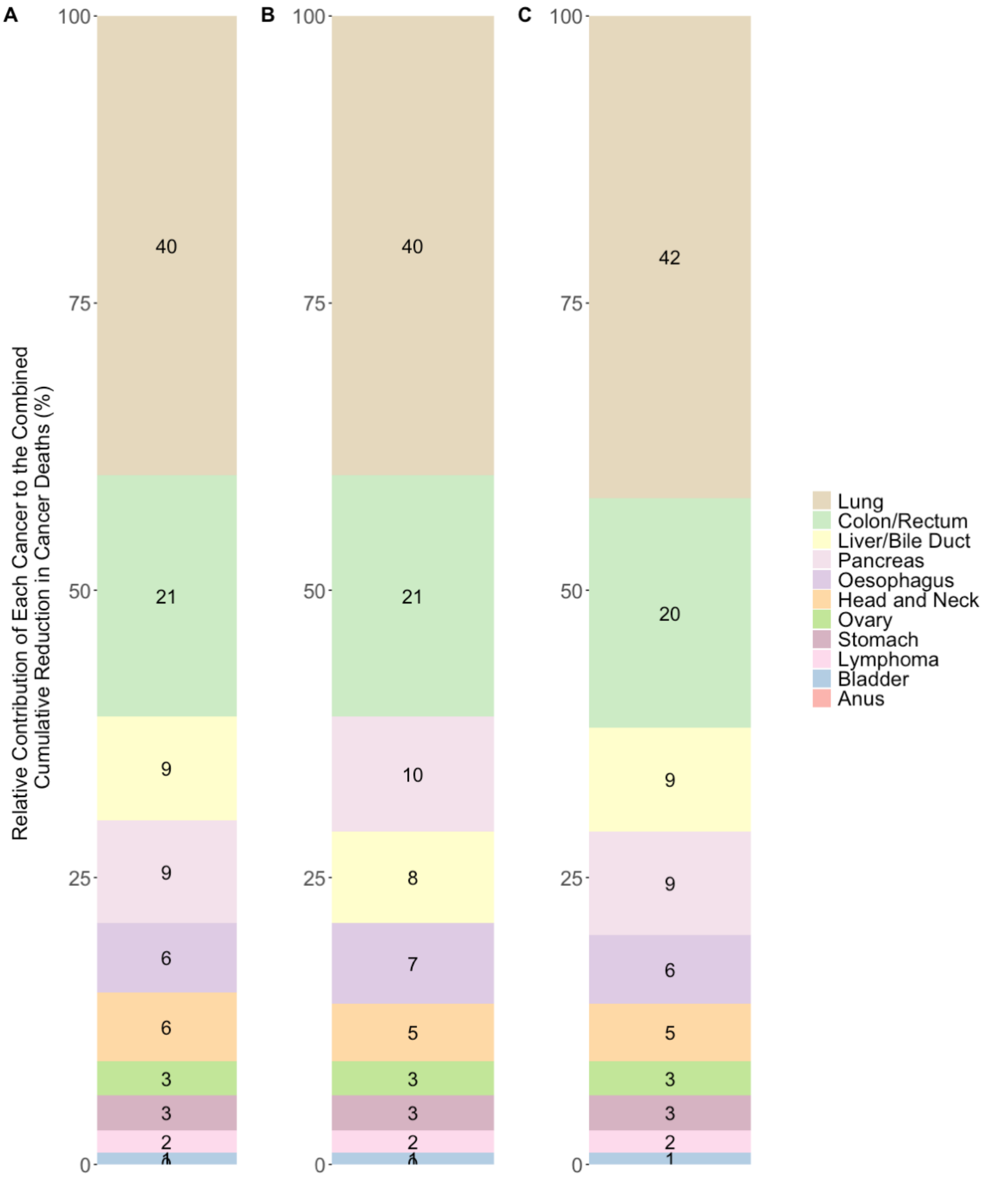


[a]Estimates assume a screening trial with 3 annual screens (months 0, 12, 24) starting at 66 years of age and 3 years of follow-up after the last (3rd) screen. Results are presented at five years after randomization under three scenarios, which we labeled based on the triplet of OMST, LMST and early-stage test sensitivity: A. (1) Fast-fast-optimistic: Overall mean sojourn time (OMST) of 1 year and late-stage mean sojourn time (LMST) of 6 months with early-stage sensitivity per cancer set to 100% of the published estimates by Klein et al[4]; B. (2) Slow-fast-conservative: OMST of 2 years and LMST of 6 months with early-stage sensitivity set to 50% of the published estimates and C.(3) Slow-slow-conservative: OMST of 2 years and LMST of 1 year with early-stage sensitivity set to 50% of the published estimates.

© 2025 Gogebakan KC et al. *JAMA Network Open*.

## eTable4. Limitations of the decision-analytic model

| Model Input/Assumption | Limitation Type | Description of Limitation |
|---|---|---|
| Overall mean sojourn time (OMST) | Data | OMST was assumed similar across cancer types due to lack of cancer-specific estimates; based on a single retrospective study. |
| Overall mean sojourn time (OMST) | Data | Specification of 1-2 years may reflect an optimistic interpretation of the single retrospective study. Appropriate values could change with advances in MCED technologies. |
| Late mean sojourn time (LMST) | Data | LMST was assumed similar across cancer types; no empirical estimates exist for variation by cancer type. |
| Stage-specific survival | Data | Based on cases diagnosed over the period 2013–2018; thus does not reflect the impact of recent therapeutic advances. Such advances can be added if utilization and benefit are known. |
| Stage-specific multi-cancer test sensitivities | Data | Prospective test sensitivities are not known. In the model estimates, we considered that early-stage sensitivity in the prospective MCED screening trial may be lower than that observed among clinically diagnosed cases (i.e., 50% of the published values in the setting of a two-year OMST). |
| Natural history model | Model structure | The natural history model assumes all cancers progress to clinical diagnosis; for cancer types without existing screening tests, the suitability of this simple model structure has not been evaluated. We note that in the setting of cancer types with existing screening tests, clinical diagnosis is defined to be diagnosis in the absence of MCED testing, thus corresponding to clinical or screen diagnosis under contemporary technologies. |
| Mortality model | Model structure | (1) The model does not account for within-stage group shifts.<br>(2) The model does not account for differing tumor subtypes.<br>(3) The model shifts cases out of late stage and into early stage preserving the relative proportion of stage III and IV cases and the relative proportion of stage I and II cases according to current partitioning of early and late stage. |

© 2025 Gogebakan KC et al. *JAMA Network Open*.

**eReferences**

1. Lange JM, Gogebakan KC, Gulati R, Etzioni R. Projecting the Impact of Multi-Cancer Early Detection on Late-Stage Incidence Using Multi-State Disease Modeling. *Cancer Epidemiol Biomarkers Prev*. 2024;**33**(6):830-7.

2. National Cancer Registration and Analysis Service. Incidence and Mortality. Available from: https://www.cancerdata.nhs.uk/incidence_and_mortality (accessed on November, 1, 2024).

3. Sasieni P, Smittenaar R, Hubbell E, Broggio J, Neal RD, Swanton C. Modelled mortality benefits of multi-cancer early detection screening in England. *Br J Cancer*. 2023;**129**(1):72–80.

4. Klein EA, Richards D, Cohn A, et al. Clinical validation of a targeted methylation-based multi-cancer early detection test using an independent validation set. *Ann Oncol*. 2021;**32**(9):1167–77.

5. Neal RD, Johnson P, Clarke CA, et al. Cell-Free DNA-Based Multi-Cancer Early Detection Test in an Asymptomatic Screening Population (NHS-Galleri): Design of a Pragmatic, Prospective Randomised Controlled Trial. *Cancers (Basel)*. 2022;**14**(19).

6. NHS England. 29 May 2024. An update on the ongoing NHS-Galleri trial. Available from: https://www.england.nhs.uk/blog/an-update-on-the-ongoing-nhs-galleri-trial/ (accessed on January 6, 2025).

© 2025 Gogebakan KC et al. *JAMA Network Open*.
