# Supplementary material for: Clinical Significance of a Multicancer Screening Trial With Stage-Based End Points
Source: JAMA Netw Open. 2025 Oct 9;8(10):e2536247. doi: 10.1001/jamanetworkopen.2025.36247 (PMC12511994; doi:10.1001/jamanetworkopen.2025.36247)
Supplement: Supplement 2. — Data Sharing Statement [file jamanetwopen-e2536247-s002.pdf]

# Data Sharing Statement

Gogebakan. Clinical Significance of a Multicancer Screening Trial With Stage-Based End Points. *JAMA Netw Open*. Published October 09, 2025.
doi:10.1001/jamanetworkopen.2025.36247

**Data**
**Data available:** No

**Additional Information**
**Explanation for why data not available:** All data used in the model were obtained from published literature and publicly available databases, all of which are cited in the manuscript. No patient level data was collected for this study.
